# Skull and Neck Lesions in a Long-Finned Pilot Whale (*Globicephala melas*): A Result of Ship Collision?

**DOI:** 10.3390/ani12182362

**Published:** 2022-09-09

**Authors:** Aage Kristian Olsen Alstrup, Christian Sonne, Melanie Brauckhoff, Jørgen Hilmer Hansen, Charlotte Bie Thøstesen

**Affiliations:** 1 Department of Nuclear Medicine & PET, Aarhus University Hospital, DK-8200 Aarhus, Denmark; 2 Department of Clinical Medicine, Aarhus University, DK-8200 Aarhus, Denmark; 3 Department of Ecoscience, Aarhus University, DK-4000 Roskilde, Denmark; 4 Department of Biology, University of Southern Denmark, DK-5230 Odense, Denmark; 5 Fisheries and Maritime Museum, DK-6710 Esbjerg, Denmark; 6 Dan ZooVet, DK-6823 Ansager, Denmark

**Keywords:** autopsy, cause of death, necropsy, ship collision, stranding, pilot whale

## Abstract

**Simple Summary:**

We performed a routine necropsy on an adult male pilot whale stranded in a heavy boat traffic area of Denmark. On its outside, there was no visible damage or alterations, and similarly, the internal organs of the thorax and the abdomen also showed no significant changes that could explain the death of the whale. But to our surprise, we found extensive internal injuries after incisions in the head and neck regions: There were multiple fractured bones, muscle trauma and extensive bleeding including a fractured occipital bone with several fragments and bone pieces deeply embedded into the whale brain—injuries that very well, but not with certainty, could be caused by a ship collision. This case demonstrates the importance of performing full necropsies of whales to rule out other causes of death. Otherwise, ship collisions may be an overlooked issue with implications for population health.

**Abstract:**

Necropsy on an adult male pilot whale stranded in Denmark in an area with heavy boat traffic revealed internal lesions in the head and neck region, while the exterior did not show any visible lesions. We found multiple fractured bones, muscle trauma and extensive hemorrhage including a fractured occipital bone with several fragments and bone pieces deeply embedded into the cerebrum of the brain. The brain was literally smashed while the third and partially fourth cervical vertebrae were almost pulverized surrounded by large amounts of blood and muscle contusion. The whale was likely killed due to a ship collision, and this particular case substantiates the value of always performing full necropsies including incisions in head and neck regions on all stranded whales—especially in areas with heavy boat traffic. This case demonstrates the importance of veterinarians performing full necropsies of whales to rule out other causes of death. Otherwise, ship collisions may be an overlooked issue having implications for population health.

## 1. Introduction

Necropsy is an important tool for the determination of cause of death in whales [1,2,3,4] and is important to help distinguish between anthropogenic and natural causes of death [2,5]. Although full necropsies are recommended, in practice faster necropsies are often performed with an exclusive focus on external injuries and internal changes in organs of the thorax and abdomen. This is especially the case with stranding of specimens of non-endangered whale species under severe autolysis [5], but the question is what is overlooked. Ship collisions have been a cause of mortality for whales since the late 1800s, and casualties have increased over the past 70 years, most likely due to the increase in vessel number, size and speed (14 kn or faster) [6,7]. Often, a whale hit by a ship is easily recognizable when stranded—particularly upon impact by the ship’s propeller that covers the skin in distinctive cut patterns [8]. Thus, hemorrhages, lacerations and even amputations are quite common after whale–ship collisions [9]. However, internal injuries (e.g., fractures of bones and organ rupture) may also occur, leaving only few external injuries and therefore, require a necropsy to help identify the cause of death. Here, we present the major necropsy findings from a stranded long-finned pilot whale (*Globicephala melas*) with no visible external lesions at all, but with major internal collision trauma in bones and muscles in the neck and head region. This case has strengthened our view on the importance of performing full necropsies on stranded whales from areas with intensive boat traffic.

## 2. Materials and Methods

On 14 December 2018, a long-finned pilot whale carcass showing moderate autolytic decay stranded on the coast of Skagen in Denmark (journal number C396). The animal was an adult male with a body weight of 1550 kg and a total length of 546 cm, corresponding to approximately 20 years [10,11]). This particular area borders the North Sea and is known for its high ship density [12], one of Europe’s highest (View Data|EMODnet Human Activities (www.emodnet-humanactivities.eu, accessed on 25 June 2022)). The carcass was transported on a truck to the Fisheries and Maritime Museum in Esbjerg (Denmark), where a necropsy was performed three days later by trained biologists, veterinarians and a conservationist. The necropsy followed the same procedures as for Hansen et al. [3]. During the necropsy, two formalin-fixed (1:10) tissue samples from the longus colli muscle next to the crushed cervical vertebrae were embedded in paraffin, sectioned at about 4 μm and H&E-stained and examined under a microscope at 50–1000× magnification. Only a few samples were examined due to the moderate autolytic decay of the whale.

## 3. Results

**Figure 1:**
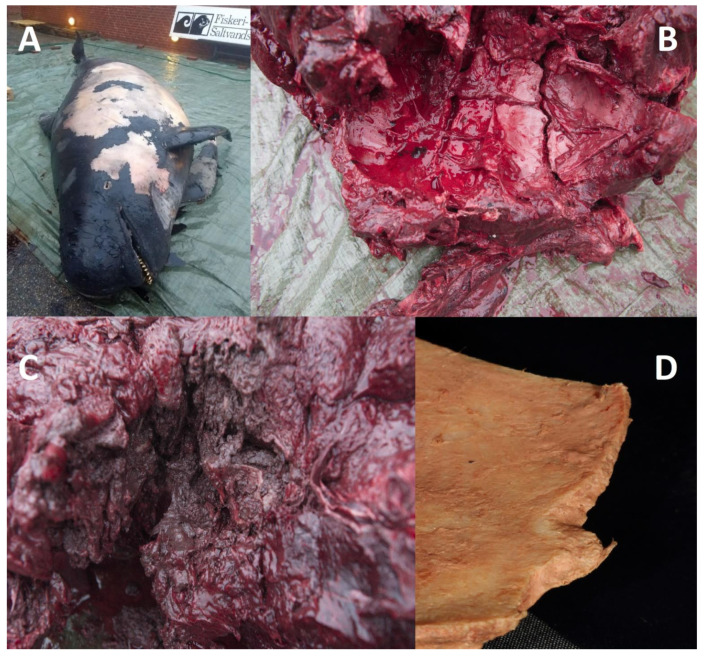
The long-finned pilot whale (*Globicephala melas*) before necropsy (**A**), extensive lesions of fractured skull bones (**B**), the traumatized neck muscles (**C**) and a fragment from the occipital bone after cleaning (**D**).

Body condition as well as blubber thickness (51–103 mm) showed that it was moderately to well-nourished prior to death. There were no penetrating wounds, lacerations or other signs of sharp trauma, which often indicate a collision with a ship or similar vessels. No signs of mechanical influence, either in the form of hemorrhages, compressions or necrosis found upon conducting parallel 3 cm-deep incisions into the blubber with a distance of 25 cm (crisscrossing) in the head and neck region. No extravasated blood was observed in the blubber. The systematic necropsy of the muscular and internal organs from the thorax and abdomen only indicated nonspecific findings, such as a few parasites (*Stenurus* spp.) in the lungs. None of these findings could be related to the cause of death. The intestines were almost empty; however, this is a normal finding in whales [13]. In addition, the stomach showed no signs of hemorrhages with no macro plastic but contained fish bones and otoliths. Heart, lungs, liver, kidney, spleen and gonad were all normal in size, firmness and shape. The dissection of the head and neck showed extensive lesions of fractured bones, muscle trauma and extensive hemorrhage. The occipital bone was fractured (area > 400 cm^2^) into multiple fragments, but with no clear signs of external hemorrhages (Figure 1). On the inside of the skull, bone pieces were embedded deeply into the cerebrum, so that the brain was partially comminuted. Similarly, the third and partially the fourth cervical vertebrae were pulverized into small fragments, while the musculatures were marked by contusions with moderate to high amounts of blood. We found bacterial and gas bubble cavities due to muscle degradation three days after the necropsy. One of the samples from the longus colli muscle consisted mainly of connective tissue, adipose tissue and a few muscle fibers dominated by diffuse hemorrhage. The other sample consisted predominantly of muscle fibers with low-grade hemorrhage. It was not possible to determine whether erythrocytes were pressed into the muscle under the pressure of impact or of merely lying on the surface upon stranding. In addition, in the samples collected from skin and blubber a focal scar tissue with leukocytic infiltration (with both neutrophil granulocytes, lymphocytes and macrophages) was observed. We furthermore found small skin lesions with mixed flora of bacteria, but these were assessed to have occurred postmortem. No pathology in the underlying blubber was observed. Organ weight and pathology findings are shown as a table in the Supplementary Materials.

## 4. Discussion

Here, we have documented and described blunt trauma in a dead pilot whale. Fractured vertebrae can occur when whales collide with fast-moving, heavy ships [14]. In this case, we identified major internal head and neck lesions that were not visible from the outside. Skull and vertebrae fractures are common findings after ship collisions, along with fractures of the ribs and mandibles [9]. In principle, the damage can also be caused by collision with large objects other than ships, including fixed installations in the sea or interactions with fishermen or other whales. However, it is less likely, considering how extensive the blunt lesions were. The histological examination was unable to determine whether the whale was dead (post mortem) or alive (ante mortem) upon the time of collision, but the hemorrhage around the vertebrae shows that the animal was probably alive at the time of impact. Our goal was to use a forensic technique to distinguish between erythrocytes pressure-injected into the musculature (indicating the whale was still alive at the time of collision) or erythrocytes simply shown as a layer on top of the musculature (indicating the whale was dead at the time of collision). Unfortunately, the tissue samples were too decayed to ascertain this hypothesis, as decomposition had been ongoing for days prior to our necropsy. However, based on body condition, the severe lesions, the moderate hemorrhage and the lack of another pathoanatomical diagnosis, it is most likely that the whale died upon collision with a ship in one of the most heavily crowded oceans in the Danish part of the North Sea [12]. Furthermore, the carrier was able to inform us that the dead whale did not sustain any damage during transportation. Unfortunately, we did not examine for edema, flocculent, degeneration of myofibrils and contraction band necrosis or fat embolism in the lungs, which may indicate whether or not the whale was alive [15,16,17]. In addition, we cannot exclude other reasons for the pathologies such as the fishing industry or attack by other toothed whales. Blunt force lesions have previously been observed after fishermen aggression in dolphins without any visible external lesions [18], and also aggressive interactions may lead to serious head lesions, such as mandible factures [19]. Killer whales may also with their snouts produce internal lesions without visible lesions in the skin [20].

According to the IUCN Red List and IWC (https://iwc.int/pilot-whale, accessed on 25 June 2022), there are no implications that the world’s population of pilot whales is being threatened by ship collisions at the moment, although cases have been reported [21,22,23,24]. However, in reported casualties of fin whales (*Balaenoptera physalus*) and right baleen whales (*Balaenidae*), up to one third of the strandings may be due to ship collisions [6]. Similarly, another nine whale species are known to collide with ships—it is particularly widespread in ships that are more than 80 m long and sail at a speed of at least 14 kn [6]. Ship collisions are, in a single study, the third most frequent cause of death in large whales in the Northwest Atlantic [22], which emphasizes the importance of being able to identify this cause of death. It is of course most problematic when whales and ships follow the same routes. The whales here are naturally at greatest risk at the surface. However, previous studies have shown that whales are more vulnerable to collision when they feed and socialize [14].

Furthermore, ship traffic affects the whales not just by colliding with them, but also causes disturbance and underwater noise [25]. It is most likely that pilot whales and other toothed whales that use echolocation are particularly at risk [26]. Although full necropsies are recommended, this is in practice not always followed for non-endangered whale species and species in severe autolysis [5]. It can be speculated that external injuries easily can be overlooked in corpses under severe autolysis. Neilson and coworkers found exclusively blunt trauma in 16 out of 21 (76%) dead whales necropsied after ship collisions [7]. In any case, this study demonstrates the importance of veterinarians always performing full necropsies, including performing incisions in the head and neck region, and thus do not settle for an examination of the whale’s exterior combined with an examination of internal organs in the thorax and abdomen. Otherwise, ship collisions may be an overlooked issue having implications for population health in that it may be underestimated in the statistics.

## 5. Conclusions

This case clearly shows that a full necropsy including an incision in the head and neck region is necessary for the identification of major internal lesions after ship collisions with whales. Surprisingly, very large internal damage can be invisible on the external surface of whales. In this particular case, the other major necropsy findings indicate that the whale may have been alive and that this collision immediately caused its death. This shows that ship as well as other collisions and traumatic events may be an overlooked issue having implications for population health.

## Data Availability

Not applicable.

## Supplementary Materials

The following are available online at https://www.mdpi.com/article/10.3390/ani12182362/s1, Table S1: Organ weight and pathology findings in a male pilot whale.
