# Supplementary material for: Skull and Neck Lesions in a Long-Finned Pilot Whale (Globicephala melas): A Result of Ship Collision?
Source: Animals (Basel). 2022 Sep 9;12(18):2362. doi: 10.3390/ani12182362 (PMC9495128; doi:10.3390/ani12182362)
Supplement: Supplementary file 1 [file animals-12-02362-s001.zip › animals-1816682-supplementary.pdf]

Supplementary file. Table S1: *Organ weight and pathology findings in a male pilot whale.*

Organ	Weight	Gross findings, findings and comments
Skin, muscles and bones	N.D.	Extensive lesions of fractured bones, muscle trauma and moderate hemorrhage were found in head and neck region. The occipital bone was fractured into multiple fragments, but with no clear signs of external hemorrhages. On the inside of the skull, bone pieces were embedded deeply into the cerebrum, so that the brain was partially wrecked. Similarly, the third and partially the fourth cervical vertebrae were pulverized, while the musculatures were marked by contusions with moderate amounts of blood. No gross further pathology observed, but focal scar tissue with leukocytic infiltration (neutrophil granulocytes, lymphocytes and macrophages) was observed. Furthermore, post mortem lesions with mixed flora of bacteria were found in the skin. No pathology in the underlying blubber observed.
Heart	4.9 kg	No pathology observed
Lungs	24.5 kg (both)	No pathology observed<br>A few nematode parasites (*Stenurus spp.?*)
Liver	13.5 kg	No pathology observed
Stomach	N.D.	No pathology or plastic observed<br>Fish bones and otoliths found
Intestines	31.7 kg	No pathology observed, and almost empty<br>No eggs found by use of the McMaster method
Kidney	4.0 kg (both)	No pathology observed
Spleen	N.D.	No pathology observed
Testes	5.0 kg (both)	No pathology observed
